# SP1‐mediated upregulation of LINGO‐1 promotes degeneration of retinal ganglion cells in optic nerve injury

**DOI:** 10.1111/cns.13426

**Published:** 2020-06-19

**Authors:** Yali Wu, Zongyi Zhan, Yadan Quan, Yangfan Yang, Xiaotao Chen, Liling Liu, Kaili Wu, Minbin Yu

**Affiliations:** ^1^ State Key Laboratory of Ophthalmology Zhongshan Ophthalmic Center Sun Yat‐sen University Guangzhou China

**Keywords:** LINGO‐1, neuroprotection, optic nerve injury, retinal ganglion cell, SP1

## Abstract

**Backgrounds:**

Insults to the axons in the optic nerve head are the primary cause of loss of retinal ganglion cells (RGCs) in traumatic, ischemic nerve injury or degenerative ocular diseases. The central nervous system–specific leucine‐rich repeat protein, LINGO‐1, negatively regulates axon regeneration and neuronal survival after injury. However, the upstream molecular mechanisms that regulate LINGO‐1 signaling and contribute to LINGO‐1–mediated death of RGCs are unclear.

**Methods:**

The expression of SP1 was profiled in optic nerve crush (ONC)–injured RGCs. LINGO‐1 level was examined after SP1 overexpression by qRT‐PCR. Luciferase assay was used to examine the binding of SP1 to the promoter regions of LINGO‐1. Primary RGCs from rat retina were isolated by immunopanning and RGCs apoptosis were determined by Tunnel. SP1 and LINGO‐1 expression was investigated using immunohistochemistry and Western bolting. Neuroprotection was assessed by RGC counts, RNFL thickness, and VEP tests after inhibition of SP1 shRNA.

**Results:**

We demonstrate that SP1 was upregulated in ONC‐injured RGCs. SP1 was bound to the LINGO‐1 promoter, which led to increased expression of LINGO‐1. Treatment with recombinant Nogo‐66 or LINGO‐1 promoted apoptosis of RGCs cultured under serum‐deprivation conditions, while silencing of SP1 promoted the survival of RGCs. SP1 and LINGO‐1 colocalized and were upregulated in ONC‐injured retinas. Silencing of SP1 in vivo reduced LINGO‐1 expression and protected the structure of RGCs from ONC‐induced injury, but there was no sign of recovery in VEP.

**Conclusions:**

Our findings imply that SP1 regulates LINGO‐1 expression in RGCs in the injured retina and provide insight into mechanisms underlying LINGO‐1–mediated RGC death in optic nerve injury.

## 1 INTRODUCTION

Retinal ganglion cells (RGC), the projection neurons of the eye, bear the responsibility of propagating visual stimuli to the brain. Under many traumatic, ischemic nerve injury or degenerative ocular conditions, such as glaucoma, the dysfunction and/or loss of RGC is the primary determinant of visual field loss and are the measurable endpoints in current research into experimental therapies.^1^, ^2^ Evaluation of the molecular mechanism underlying RGC neuropathy is important as it may facilitate development of novel therapeutics that ameliorate glaucoma by promoting the survival and axonal regeneration of RGCs.

The lack of cellular and axonal regeneration in the event of neuronal injuries is due to myelin‐associated inhibitory factors.^3^, ^4^, ^5^ The leucine‐rich repeat and immunoglobulin‐like domain‐containing protein 1, LINGO‐1, is expressed by neurons and oligodendrocytes in the central nervous system (CNS) and is an essential component of the NgR/p75 or NgR/Troy signaling complex, which binds to myelin‐associated inhibitory ligands.^6^, ^7^ LINGO‐1 is reported to negatively regulate myelination and neurite extension and to mediate breakdown of the neuronal growth cone.^6^, ^8^, ^9^ Importantly, its expression is elevated in patients with various degenerative diseases and those with CNS injuries,^7^, ^10^, ^11^, ^12^ suggesting the potential pathological role of LINGO‐1 in CNS diseases. Moreover, the LINGO‐1 gene is related to risk for neuronal apoptosis in patients with neurodegenerative diseases,^13^, ^14^ implying that modulation of myelin inhibitor signaling may promote the survival of neurons and myelination of oligodendrocytes after injury. Inhibition of LINGO‐1 has neuroprotective effects in models of several CNS diseases and injuries. In two previous studies, a LINGO‐1 antagonist significantly increased oligodendrocyte and neural survival, and promoted axonal regeneration and functional recovery after spinal cord injury.^15^, ^16^ In a study that used a mouse model of Parkinson's disease, survival of dopaminergic neurons increased and behavioral abnormalities were reduced in LINGO‐1–knockout mice compared to wild‐type mice.^12^ In addition, LINGO‐1 antagonists have neuroprotective effects against injury‐induced apoptosis in cultured neurons.^12^, ^17^, ^18^ We previously reported that in an optic nerve crush (ONC) model, inhibition of LINGO‐1 by RNA interference promoted regeneration of the optic nerve and the survival of RGCs.^19^ Although inhibition of LINGO‐1 promotes the survival of neurons and RGCs, the underlying mechanism is unclear.

Upon axonal injury, transcription factors in neurons are activated, resulting in a cascade of changes in the transcriptome and priming of the degeneration and regeneration pathways.^20^ SP1, a multifunctional zinc finger transcription factor that binds to GC‐rich motifs in DNA, is implicated in stress‐related apoptosis of neurons and the pathogenesis of a variety of degenerative diseases.^21^, ^22^, ^23^ However, little is known of the role of SP1 in the regulation of retinal neuropathy. Here, we demonstrate that SP1 regulates the expression of LINGO‐1 and contributes to LINGO‐1–mediated death of RGCs in the ONC‐injured retina. These findings provide insight into the mechanism of the death of RGCs in patients with glaucoma and imply that SP1 and LINGO‐1 may be potential therapeutic targets in neuroprotection strategies for glaucoma.

## 2 MATERIALS AND METHODS

### 2.1 Animals and ethics statement

A total of 52 male Sprague Dawley rats (weight, 180‐200 g; age, 6‐8 weeks) and 40 newborn rats (age, 3‐5 days) were maintained in the Ophthalmic Animal Laboratory of Zhongshan Ophthalmic Center. All procedures involving animals were conducted in accordance with the ARVO Statement for the Use of Animals in Ophthalmic and Vision Research. All experimental procedures were approved by the institutional animal care and use committee of Zhongshan Ophthalmic Center (Permit SYXK 2018‐025). All manipulations were performed with rats under general anesthesia with 2%‐3% inhaled isoflurane, and the eyes of the rats were administered topical 0.5% Alcaine eye drops (Alcon) prior to surgery, experimentation, and electrophysiology examination.

### 2.2 Optic nerve crush model

Optic nerve crush injury was performed as described previously.^19^, ^24^ In brief, after general anesthesia, a lateral canthotomy was performed on the temporal conjunctiva of the right eye of the rat using conjunctival scissors, the lateral rectus muscle was detached, and the optic nerve was exposed under a binocular surgical microscope. A Dumont #5 clip (World Precision Instruments) was applied to the optic nerve 2 mm behind the posterior eye pole for 5 seconds to provide a consistent clamping force and so ensure the reproducibility of the injury. The left eyes of the rats underwent sham surgery, which entailed exposure of the optic nerve but no ONC injury.

### 2.3 Constructs and dual‐luciferase reporter assays

The LINGO‐1 promoter fragment, comprising nucleotides −2104 to +121 bp of the LINGO‐1 5′‐flanking region relative to the transcription start site, was amplified by PCR (forward primer, 5′‐AGGTACCGAGCTCTTACGCGT‐AGTGT‐3’; reverse primer, 5′‐CAGTACCGGAATGCCAAGCTTGCTGGCT‐3’) and fused upstream of the luciferase reporter in the pGL3‐Basic vector to generate the LINGO‐luciferase (Luc) reporter. Deletion reporter constructs of the 5′‐flanking region were generated by PCR using LINGO‐1–Luc as the template and a common reverse primer. The forward primers were as follows: 5′‐GAAGGCGAACAAGGCACTG‐3′ for LINGO‐1 (−1268 to +121)–Luc, 5′‐AGCTGAGCCCAGACTAAG‐3′ for LINGO‐1 (−789 to +121)–Luc, 5′‐ATGGCAGTGTGCAGTGAC‐3′ for LINGO‐1 (−383 to +121)–Luc, 5′‐CTCCCTGGCTCGCTGCTC‐3′ for LINGO‐1 (−122 to +121)–Luc. All PCR products were subcloned into the *Xho*I/*Hind*III restriction enzyme sites in the pGL3‐Basic plasmid. For dual‐luciferase reporter assays, cells were cotransfected with 1 µg *firefly* luciferase plasmid harboring the promoter fragments and 100 ng of the *Renilla* luciferase reporter plasmid pRL‐TK. The cells were harvested at 36 hours after transfection, and *Firefly* activity and *Renilla* luciferase activity were measured using the Dual‐Glo Luciferase Reporter Assay System (Promega). *Firefly* luciferase activity was normalized to that of *Renilla* luciferase and is presented as relative luciferase units.

### 2.4 Primary culture of RGCs and survival assays

Primary RGCs were isolated and purified by immunopanning.^25^ Briefly, the retinas of 3‐day‐old SD rats were triturated and digested with 5 mg/mL papain. The dissociated cell suspension was incubated on a panning plate coated with goat anti‐rabbit IgG to remove macrophages. Next, the Thy1.1‐positive RGCs were purified using a second panning plate coated with goat anti‐mouse IgM and mouse anti‐Thy1.1 antibodies (Invitrogen). The detached RGCs were seeded at a density of ~ 1.5 × 10^4^/mm^2^ and cultured in Dulbecco's modified Eagle's medium (DMEM) containing 10% fetal bovine serum (FBS), BDNF (50 ng/mL; Peprotech), CNTF (10 ng/mL; Peprotech), and forskolin (5 ng/mL; Sigma‐Aldrich) in culture slides precoated with poly d‐lysine and laminin (Sigma‐Aldrich). For survival assays, SP1‐shRNA was transfected into cells using Lipofectamine RNAiMAX as recommended by the manufacturer. At 48 hours after transfection, the cells were treated with or without recombinant Nogo‐66 (50 μg/mL; R&D Systems) or LINGO‐1 (100 μg/mL; LifeSpan) in the presence of absence of SP1‐shRNA. After incubation for 12 hours, complete medium was replaced with DMEM without serum to induce apoptosis. Cell cultures with serum were performed in parallel as controls.

### 2.5 Quantitative reverse transcription PCR

Total RNA was extracted from retina tissues using TRIzol reagent (Invitrogen). RT‐qPCR was performed using PrimeScript RT Master Mix and SYBR Premix Ex Taq II (Tli RNaseH Plus; TaKaRa Bio) according to the manufacturer's instructions. The sequences of the primers used for qPCR were as follows: LINGO‐1, 5′‐CTTCCCCTTCGACATCAAGAC‐3′ and 5′‐AAGACGGACCACGACGAC‐3′; β‐actin, 5′‐TCACCCACACTGTGCCCAT‐3′ and 5′‐TCTTTAATGTCACGCACGATT‐3′; SP1, 5′‐TCCAGACCATTAACCTCAGTGC‐3′, and 5′‐ACCACCAGATCCATGAAGACC‐3′.

### 2.6 Western blotting

Retinas were homogenized, and total protein was extracted using a Protein Extraction Kit (Beyotime Biotechnology). The total protein samples were separated by sodium dodecyl sulfate‐polyacrylamide gel electrophoresis, electro‐transferred to a nitrocellulose membrane, and exposed to anti‐SP1 (1:500; Millipore) and anti‐LINGO‐1 (1:500; Upstate) antibodies. Next, the membrane was incubated with a horseradish peroxidase–conjugated secondary anti‐rabbit antibody (CST); β‐actin served as the loading control. The protein bands were detected by enhanced chemiluminescence (Pierce).

### 2.7 Immunofluorescence assay

After cardiac perfusion with 0.9% saline, the eyes of the rats were collected, fixed in 4% paraformaldehyde for 12 hours, and cryoprotected in 30% sucrose for 12 hours at 4°C. The eyes were next embedded in optimal cutting temperature medium and sectioned (10 μm thickness). The sections were permeabilized with 0.3% Triton X‐100 and blocked with 10% goat serum for 45 minutes. The slides were incubated with an anti‐Sp1 (1:100; Millipore) or anti‐LINGO‐1 (1:100; Upstate) primary antibody overnight at 4°C, washed three times, and incubated with the secondary antibodies (1:500; Molecular Probes) for 1 hours at room temperature. Nuclei were stained with 4′, 6‐diamidino‐2‐phenylindole, and the slides were mounted and visualized under a confocal microscope (LSM 780; Carl Zeiss).

### 2.8 Terminal deoxynucleotidyl transferase dUTP nick‐end labeling staining

Terminal deoxynucleotidyl transferase dUTP nick‐end labeling (TUNEL) staining was performed using an in situ cell detection kit according to the manufacturer's instructions (Roche). Briefly, cultured RGCs were fixed in 4% PFA for 15 minutes at 4°C, blocked using 1% donkey serum, and permeabilized with 0.1% Triton at room temperature for 20 minutes. TUNEL detection solution was added, and the samples were incubated for 50 minutes at 37°C and costained with DAPI. TUNEL‐positive nuclei were quantified using ImageJ software. Finally, TUNEL‐positive cells were enumerated in high‐power fields of view of three wells per treatment, and the mean was calculated.

### 2.9 Intravitreal injections

After general anesthesia and ocular surface anesthesia, intravitreal injections were performed 2 mm behind the limbus using a Hamilton micro‐injector with a 30‐gauge needle. Five microliters of AAV2‐SP1 shRNA (1 × 10^12^ GC/mL; GeneChem) was injected into the vitreous cavity of the rats in the experimental group at 14 days before ONC injury, avoiding damaging the lens and fundus hemorrhage and ensuring that the intraocular pressure did not increase markedly. The sequence of the SP1 shRNA was 5′‐GCAACAUGGGAAUUAUGAATT‐3′.

### 2.10 Enumeration of RGCs in flat‐mounted retinas

Rats were sacrificed and eyes were collected, fixed with 4% PFA for 2 hours. The intact retinas were separated, and five radial incisions were made to create a petal shape. The retinas were permeabilized with 2% Triton X‐100, blocked with 5% goat serum for 4 hours, and incubated with an anti‐RBPMS antibody (1:100; ProteinTech) at 4°C in a shaker overnight. After three times of washes, the retinas were incubated with a secondary antibody conjugated to Alexa Fluor 488 (1:500; Invitrogen) for 4 hours. The retinas were transferred to glass slides using an enlarged open‐pad tube, flattened, blotted dry, stained with DAPI, mounted, and RBPMS‐positive RGCs were visualized under a confocal microscope (LSM 780; Carl Zeiss). RGCs were enumerated as described previously.^26^ Fifteen regions (0.055 mm^2^) were evaluated at 1.5, 2.5, and 3.5 mm from the optic nerve head across the five petals for each retina. For each petal, images of three regions representing the peripheral, medial, and central parts of the retina were acquired.

### 2.11 Recording of the visual evoked potential

Before assessment of visual evoked potentials (VEPs), the rats were dark‐adapted for > 2 hours. The rats were anesthetized by intraperitoneal injection of 10% chloral hydrate. VEPs were evaluated using a Roland RETI‐scan system (Roland Consult) for a full‐field flash stimulator. The stimulus intensity was 5 dB (9.49 cd × s/m^2^), the stimulation frequency was 1.0 Hz, the passband was 0.5‐50 Hz, and the stimulation frequency was 100. For quantitative analyses, the VEP system detection index was the N1 wave, P1 wave latency (ms), and N1‐P1 wave amplitude (μv). A visual stimulus of 1 Hz white light (9.49 c × s/m^2^) was generated by a full‐field Ganzfeld stimulator under dark‐adapted conditions. The amplitude of N1‐P1 and the latency of the N1 and P1 peaks were measured using Roland software (Roland Consult). The amplitude of N1‐P1 was determined as the interval from the trough of the first negative peak after light onset (N1) to the peak of the first positive wave (P1). The latency of the N1 and P1 waves was measured from light onset to the peak of N1 or P1.

### 2.12 Optical coherence tomography imaging

To assess ONC‐induced changes in peripapillary retinal nerve fiber layer thickness (RNFLT), we performed volume scans using a noninvasive high‐resolution SD‐optical coherence tomography (OCT) instrument (Spectralis HRA + OCT, Heidelberg Engineering) at baseline and 2, 7, 14, and 21 days after surgery as described previously.^26^ The rats were anesthetized by intraperitoneal injection of 10% chloral hydrate. Next, they were placed on a freely rotating platform to align the eye with the OCT lens. SD‐OCT uses a super‐radiative light‐emitting diode with a wavelength of 870 nm as a low‐coherence light source. The recorded B‐scan consisted of 1536 A‐scans acquired at 40 000 per second. Fourier analysis techniques were used to perform postpole asymmetry analyses centered on the optic nerve head, including the mean and fan‐shaped (superior, inferior, nasal, and temporal) RNFL. The eye‐tracking technology of SD‐OCT eliminates scan artifacts and enables precise and reproducible positioning. Each eye was scanned at least three times with a signal quality of >20 dB. Follow‐up scans were performed at the indicated time points using the eye‐tracking technique to evaluate the thickness of the retinal layer at the same location as the baseline scan.

### 2.13 Statistics

All experiments were performed in at least triplicate biological repeats. Data are presented as means ± standard deviations (SDs). Statistics was performed using the statistical package for the social sciences (SPSS) software. A *P* value <.05 was considered indicative of significance. Kolmogorov‐Smirnov tests were used to assess data distribution for normality. The Student *t* test, one‐way analysis of variance (ANOVA), two‐way ANOVA, or repeated measure ANOVA was used to compare differences between groups.

## 3 RESULTS

### 3.1 SP1 is upregulated in RGCs isolated from ONC‐injured retina

**FIGURE 1:**
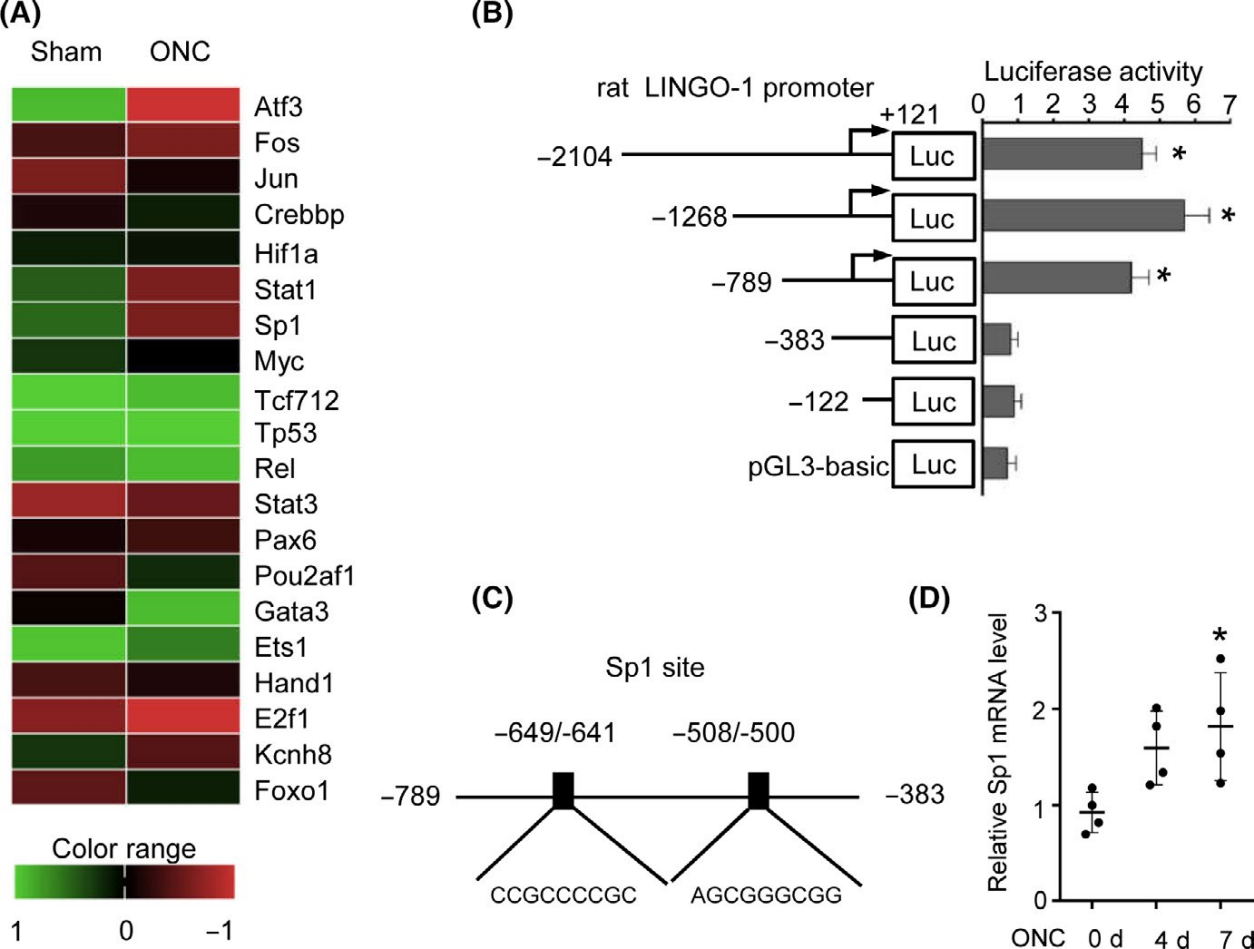
SP1 is upregulated in RGCs isolated from ONC‐injured rat retina. A, Heat map of expression of TFs in RGCs from sham and ONC rats after 4 d. B, Functional serial deletion analyses of the LINGO‐1 promoter. LINGO‐1 promoter 5′ sequential deletion constructs were created and fragments of the LINGO‐1 promoter of different lengths were cloned into the pGL3‐Basic plasmid. HEK293 cells were cotransfected with the LINGO‐1 promoter constructs and pCMV‐luc, incubated for 24 h, and luciferase activity was assayed and normalized to pGL3‐Basic (n = 3, *P < .05, and compared to pGL3‐basic control by ANOVA with the LSD multiple comparison test. C, Two putative SP1 binding sites at nucleotides −649 to −641 and −508 to −500 of the LINGO‐1 promoter. D, Relative mRNA level of SP1 in RGCs at 0, 4, and 7 d post‐ONC by qRT‐PCR (n = 4, means ± SD, compared to 0 d by ANOVA with the Tukey multiple comparison, *P < .05)

To determine the transcriptional control responsible for increased LINGO‐1 expression in RGCs, we used a gene‐expression microarray to identify genes that mediated RGC death. The efficacy of RGCs isolation was verified by cell morphology and flow cytometry with CD90.1 and CD48 labeling (Figure S1A,B). The expression profile of the transcriptional factors of ONC‐injured RGCs and sham RGCs was compared, and markedly unregulated (≥ 1.5‐fold) transcriptional factors were considered candidate genes, including ATF3, STAT1, SP1, and KCNH8 (Figure 1A).

Next, we determined the region responsible for transcriptional regulation of LINGO‐1 and the related transcriptional factors. A series of fragments of luciferase reporter constructs containing deletions of the LINGO‐1 5′‐flanking region were transfected into cells, and luciferase activity was assayed. One deletion fragment (−789 to +121 bp) showed dramatically higher promoter activity than pGL3‐Basic, similar to the activity of the full‐length fragment (−2104 to +121 bp). By contrast, luciferase activity was almost completely abolished by a different fragment (−383 to +121 bp). Thus, the region responsible for transcriptional control of LINGO‐1 was located at nucleotides −789 to −383 (Figure 1B). Computational analyses using PromoterInspector revealed two putative SP1 binding sites, CCGCCCCGC (−649 to −641 bp) and AGCGGGCGG (−508 to −500 bp) (Figure 1C). qRT‐PCR analyses showed that the SP1 mRNA level was significantly increased in injured RGCs compared to control RGCs (7 days post‐ONC) (Figure 1D). Taken together, these data indicate that upregulation of SP1 modulates the expression of LINGO‐1 at the transcriptional level.

### 3.2 Regulation of LINGO‐1 expression by SP1 and inhibition of SP1 attenuate LINGO‐1–mediated death of RGCs in vitro

**FIGURE 2:**
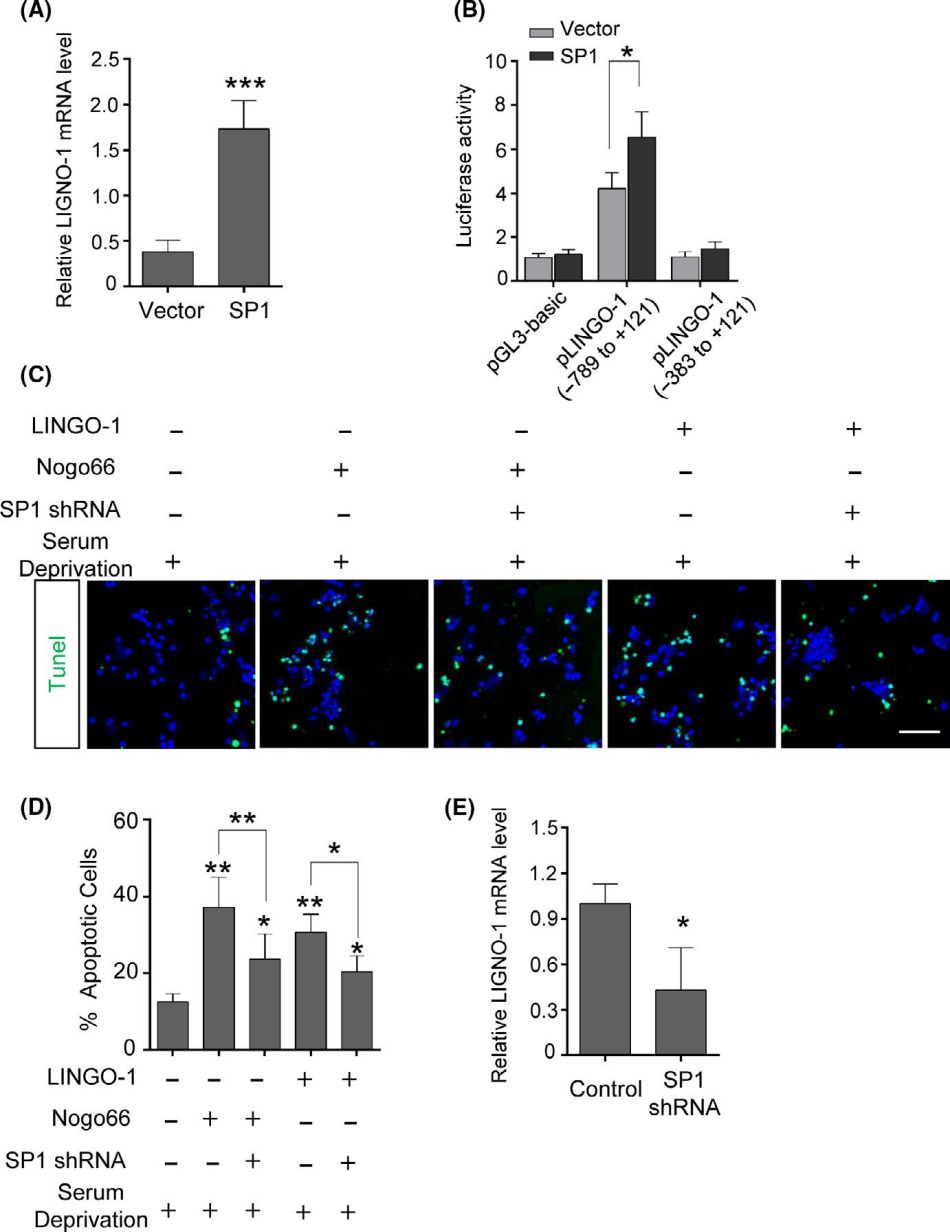
Regulation of LINGO‐1 by SP1 and inhibition of SP1 attenuated LINGO‐1–mediated RGC death in vitro. A, RT‐qPCR analysis showed that overexpression of SP1 increased LINGO‐1 expression in HEK293 cells (n = 4, means ± SD compared to vector by Student's *t* test, ***P < .001). B, Luciferase reporter analysis showed that SP1 increased the promoter activity of pLINGO‐1 (n = 3, means ± SD compared to vector by two‐way ANOVA with Bonferroni test, *P < .05). C, Inhibition of SP1 promoted survival of RGCs in the presence of LINGO‐1 in vitro. Representative images of the effects of Nogo66 or LINGO‐1 on serum‐deprived RGCs with or without SP1‐shRNA. Cell viability was assessed by TUNEL staining. Scale bar, 50 µm. D, Statistical analyses of results in (C) and percentage of TUNEL‐positive RGCs (n = 5, means ± SD by ANOVA with the Tukey multiple comparison, *P < .05, **P < .01). E, LINGO‐1 mRNA level was determined by RT‐qPCR after SP1 inhibition (n = 5, means ± SD, compared to control RGCs by Student's *t* test, *P < .05)

To investigate the role of SP1 in the regulation of LINGO‐1 expression, we transfected HEK293 cells with pCGN‐SP1 or control vector and quantified the LINGO‐1 mRNA level by qRT‐PCR. SP1 overexpression resulted in a significantly higher LINGO‐1 mRNA level compared to the control (Figure 2A). To determine the effects of SP1 on the LINGO‐1 promoter, SP1‐overexpressing or control HEK293 cells were cotransfected with promoter‐reporter fragments containing the two putative SP1‐binding sites (−789 to +121 bp) or no SP1‐binding site (−383 to +121 bp) and the promoter activity was measured. pLINGO‐1 (−789 to +121 bp) activity was markedly increased in SP1‐overexpressing cells, whereas that of a different pLINGO‐1 fragment (−383 to +121 bp) was considerably suppressed (Figure 2B).

Because LINGO‐1 is implicated in potentiating neuronal apoptosis under serum‐deprivation conditions,^18^, ^27^, ^28^, ^29^ and the LINGO‐1 receptor complex can be activated by its agonist Nogo‐66^28^ or by self‐interaction *in trans*,^30^ next, we examined whether inhibition of SP1 would have a neuroprotective effect against LINGO‐1‐mediated apoptosis of RGCs. Primary RGCs were cultured under serum‐deprivation conditions and treated with Nogo‐66 or LINGO‐1 with or without the SP1 shRNA. Cell viability was evaluated by TUNEL staining. Compared to the serum‐deprivation control, the number of TUNEL‐positive cells in serum‐deprivation cultures treated with Nogo‐66 was significantly increased. The number of apoptotic cells was markedly decreased in SP1 shRNA‐transfected, serum‐deprivation cultures treated with Nogo‐66 (Figure 2C,D). Similar trends were observed when serum‐deprivation cultures were treated with LINGO‐1 without or with the SP1 shRNA (Figure 2C,D). In addition, the LINGO‐1 expression was found decreased after SP1 inhibition (Figure 2E). Collectively, these results demonstrate that SP1 upregulates LINGO‐1 expression and in so doing contributes to LINGO‐1–mediated death of neurons.

### 3.3 LINGO‐1 and SP1 colocalize in sham and ONC‐injured retinas

**FIGURE 3:**
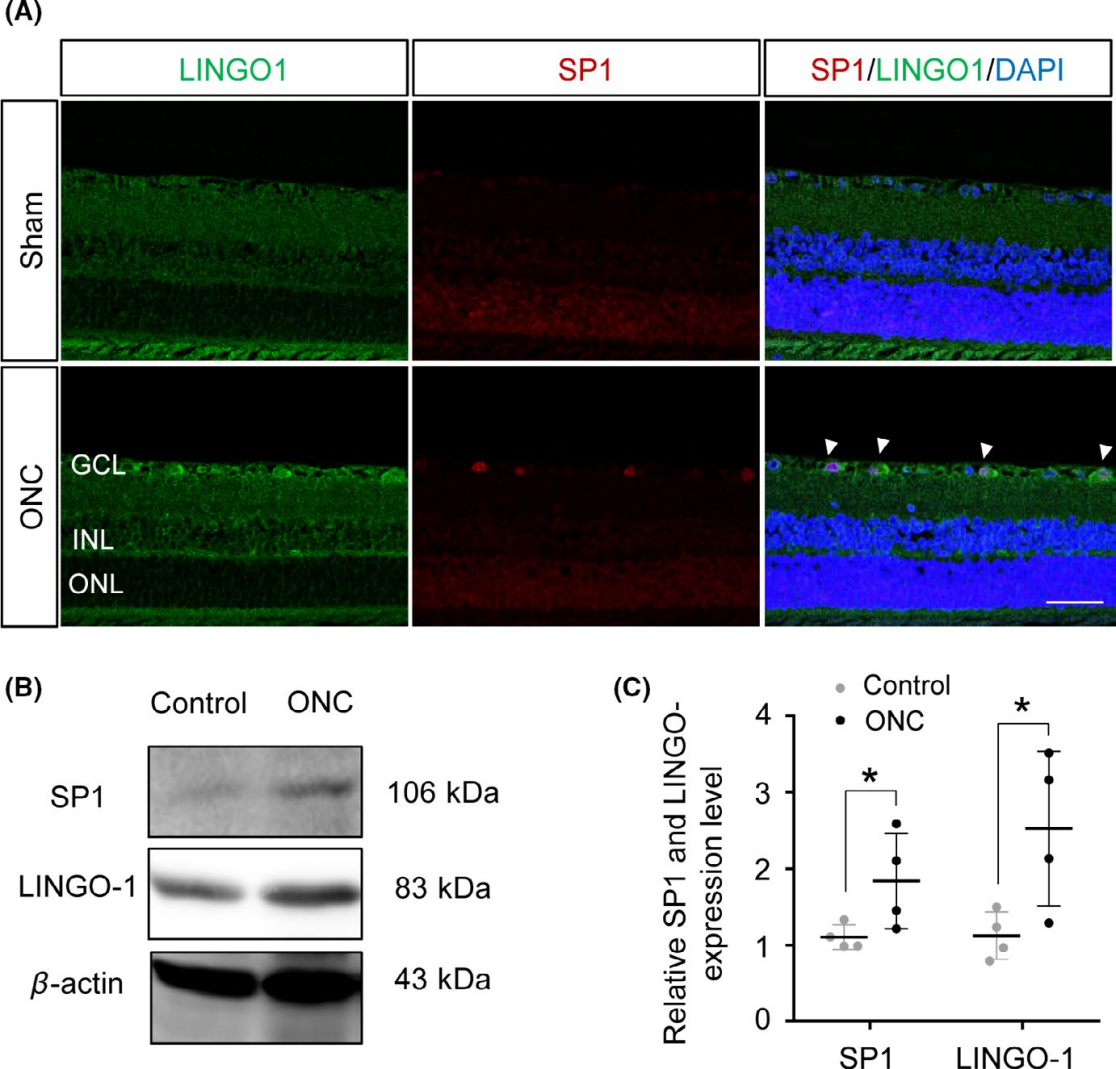
Expression of LINGO‐1 and SP1 was increased in sham and ONC‐injured retinas. A, Representative immunofluorescence staining of LINGO‐1 and SP1 in sham and ONC retinas at 8 d post‐ONC. Scale bar, 50 μm. GCL, ganglion cell layer; INL, inner nuclear layer; and ONL, outer nuclear layer. B, Immunoblotting of SP1 and LINGO‐1 in retinas at 8 d post‐ONC. C, Densitometric analyses of immunoblots of SP1 and LINGO‐1 (n = 4, means ± SD, compared to control retina by Student's *t* test, *P < .05)

To examine the association between LINGO‐1 and SP1 in the ONC‐injured retina further, double immunofluorescence staining of LINGO‐1 and SP1 was performed. The control RGCs exhibited low LINGO‐1 and SP1 expression; in contrast, LINGO‐1 and SP1 expression was considerably higher in ONC‐injured RGCs. In addition, LINGO‐1 and SP1 were colocalized in the RGCs (Figure 3A). Western blotting analyses showed that SP1 and LINGO‐1 were upregulated 1.7‐ and 2.5‐fold, respectively, in the ONC‐injured retina compared to the control (Figure 3B,C). Our results showed that the expression pattern of SP1 paralleled that of LINGO‐1 in ONC‐injured RGCs, further implying that SP1 may regulate LINGO‐1 in retinopathy.

### 3.4 Inhibition of SP1 downregulates LINGO‐1 expression in the ONC‐injured retina

**FIGURE 4:**
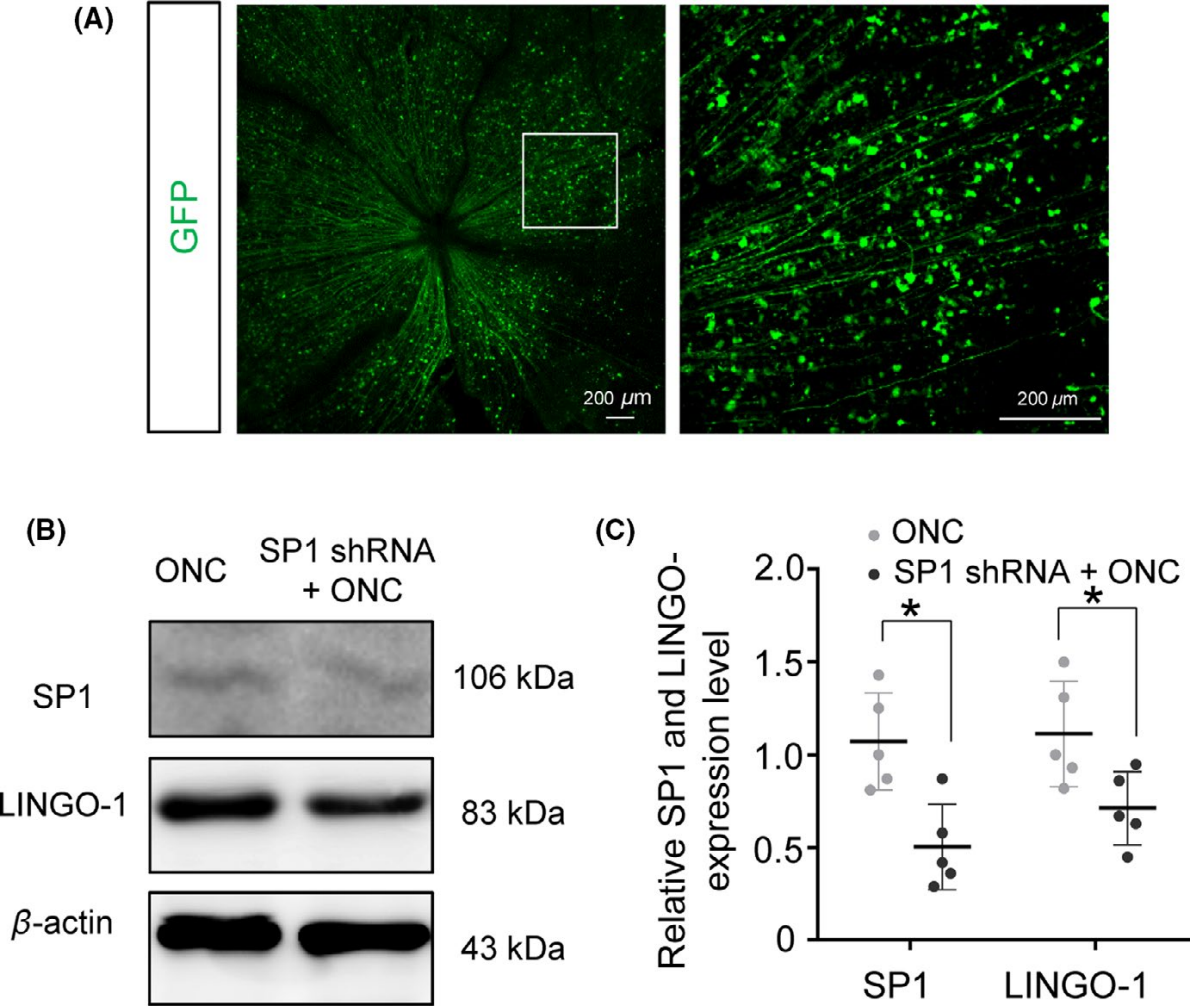
Transfection efficiency of AAV2‐SP1‐shRNA in vivo. A, Representative confocal images of AAV‐mediated SP1 transfection in RGCs in the rat retina (the boxed region is shown at higher magnification in the right panel). B, Representative images of SP1 and LINGO‐1 expression in the ONC‐injured retina with or without injection of AAV2 SP1‐shRNA. C, Quantification analysis of SP1 and LINGO‐1 expression in the ONC‐injured retina with or without injection of AAV2 SP1‐shRNA. (n = 5, means ± SD, compared to ONC retina by Student's *t* test, *P < .05)

Inhibition of LINGO‐1 promotes the survival of RGCs following ONC,^19^, ^31^ implying that LINGO‐1 is essential for the ONC‐induced loss of RGCs. Given that SP1 regulates LINGO‐1 in the retina, thereby potentiating LINGO‐1–mediated death of neurons in vitro, we investigated the effects of inhibition of SP1 on the survival of RGCs after injury to the optic nerve. To this end, we performed intravitreal injection of AAV‐SP1 shRNA to knock down SP1 expression in RGCs. Transfection efficiency was confirmed by the expression of GFP in whole‐mount retinas (Figure 4A). Our results showed that silencing SP1 abolished the ONC‐induced increase of LINGO‐1 expression (Figure 4B,C).

### 3.5 Inhibition of SP1 enhanced the survival of RGCs after ONC in vivo

**FIGURE 5:**
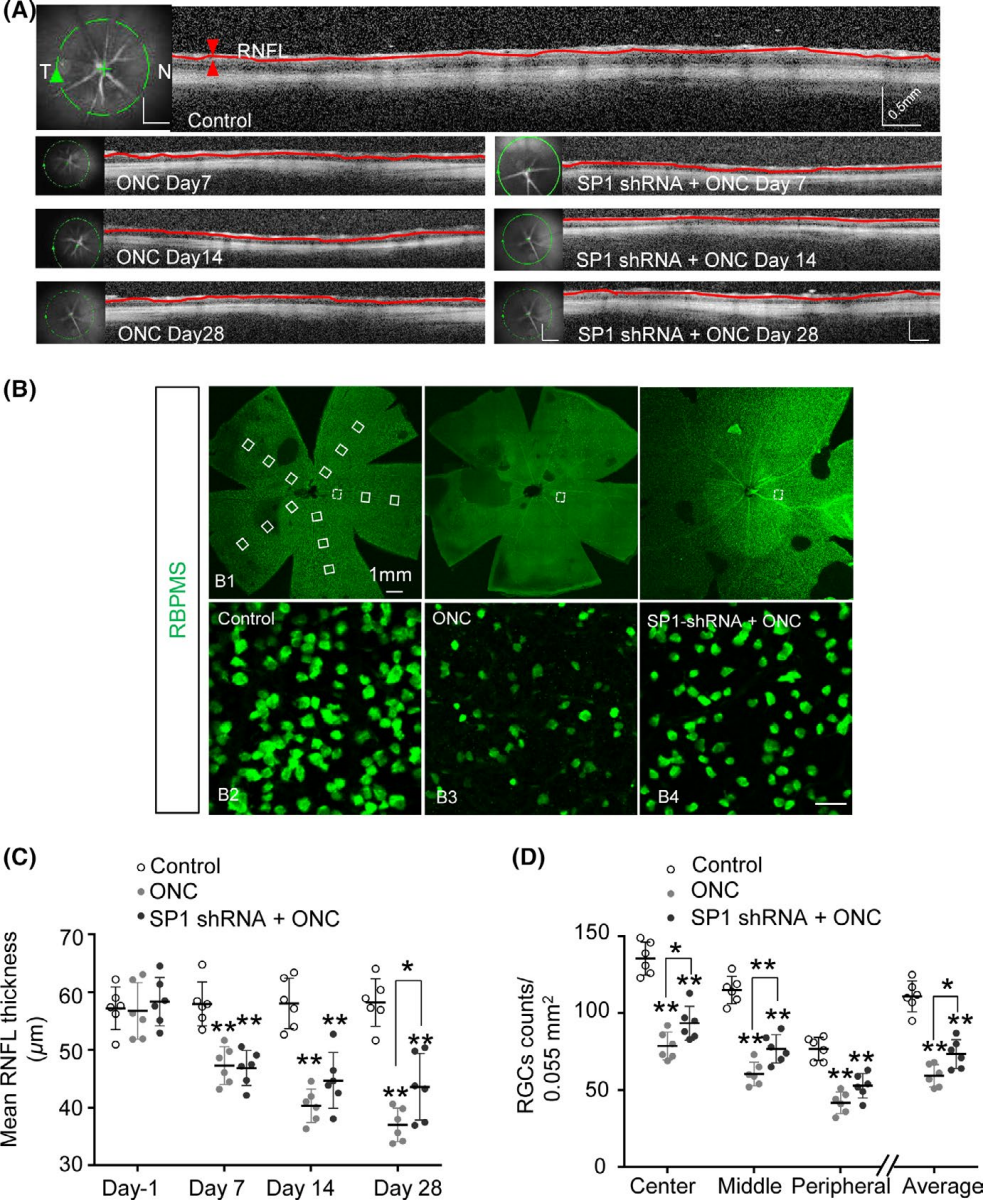
Inhibition of SP1 maintained RNFLT and promoted RGC survival after optic nerve injury. A, OCT was performed to assess RNFLT at 7, 14, and 28 d post‐ONC. RNFL images were taken of a circle (green line) centered on the optic nerve head; the full length of the RNFL is indicated by the red line. T and N, temporal and nasal regions of the retina. B, Whole‐mount retinas were immunostained with anti‐RBPMS and RGCs were enumerated. (B1) Sample areas (0.055 mm^2^) in the center, middle, and peripheral regions across five petals of the retina are boxed. Representative images of RBPMS counts of the middle regions of control (B2), ONC (B3), and SP1‐shRNA + ONC (B4) retinas. C, Mean thickness of full‐length RNFL at the indicated time points post‐ONC (n = 6, means ± SD, by RM one‐way ANOVA with the Greenhouse‐Geisser correction and Tukey multiple comparison test, *P < .05. D, Number of surviving RGCs at 28 d post‐ONC in the center, middle, and peripheral regions, and the average of all regions (n = 6, mean ± SD, by two‐way ANOVA with Bonferroni test, *P < .05, **P < .01). RNFL, retinal nerve fiber layer; OCT, optical coherence tomography

To examine the neuroprotective effects of inhibition of SP1, we monitored changes in RNFLT by OCT, a noninvasive method of assessing degenerative changes in converging axons of RGCs. The average RNFLT was 1.5 mm from the center of the optic nerve head (Figure 5A). The RNFL was significantly thinner in the ONC‐injured retina than the sham control retina at 7, 14, and 28 days. Although the RNFLTs of SP1‐transfected ONC‐injured retinas did not differ significantly from those of ONC‐injured retinas at 7 and 14 days, the reduction in RNFLT reached statistical significance at 28 days, and the ONC injured, SP1 shRNA‐treated group have a thicker RNFL than ONC‐injured group (Figure 5C).

We enumerated RGCs in the retina by counting RBPMS‐positive cells. The sampling zone (0.055 mm^2^) (Figure 5B) encompassed the central, medial, and peripheral regions of the whole‐mount retina. As we reported previously,^19^ there was considerable loss of RGCs in all regions of the retina at 28 days post‐ONC compared to the sham control. Survival of RGCs in the central, medial regions of the retina was promoted by SP1 transfection. The mean number of RGCs was also higher than the ONC group (Figure 5D). Therefore, inhibition of SP1 had a neuroprotective effect and preserved the structure of RGCs in ONC‐injured retinas.

### 3.6 Effect of inhibition of SP1 on the VEP after ONC

**FIGURE 6:**
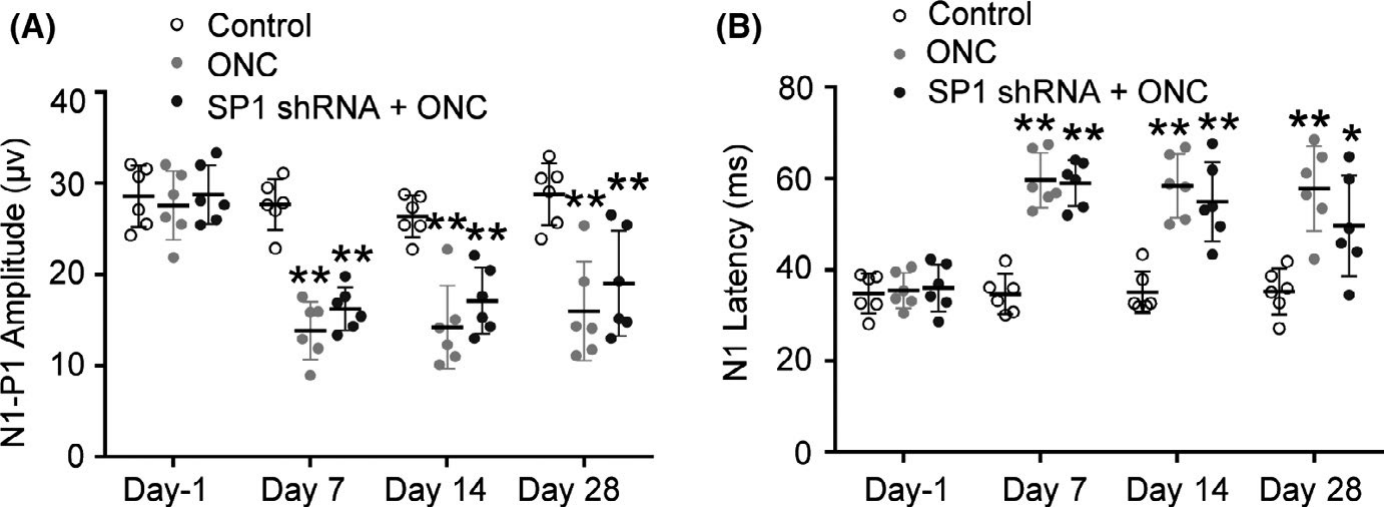
Effect of inhibition of SP1 on F‐VEP in rats after optic nerve injury. A, Average N1‐P1 amplitude at 1 d pre‐ONC, and at 7, 14, and 28 d post‐ONC. There were no significant differences between the ONC group and SP1 shRNA group at any time point (n = 6, means ± SD, by RM one‐way ANOVA with the Greenhouse‐Geisser correction and Tukey multiple comparison test, **P < .01). B, Average N1 latency component at 1 d pre‐ONC, and at 7, 14, and 28 d post‐ONC. There were no significant differences between the ONC group and SP1 shRNA group at any time point (n = 6, means ± SD, by RM one‐way ANOVA with the Greenhouse‐Geisser correction and Tukey multiple comparison test, *P < .05, **P < .01)

The preservation of RGC structure motivated us to evaluate the functional recovery of the visual circuits after injury to the optic nerve by assessing the VEP at 1‐day pre‐ONC (baseline) and at 7, 14, and 28 days post‐ONC. Impairment of visual function after ONC was evidenced by reduced amplitude of N1‐P1 waves and prolonged latency of N1 waves (Figure 6A). However, no significant differences were observed in N1‐P1 amplitude and N1 latency did not differ significantly between the ONC group and the SP1 transfection + ONC group at 7, 14, and 28 days (Figure 6B). Therefore, inhibition of SP1 did not prevent or attenuate ONC‐induced visual function impairment.

## 4 DISCUSSION

Death of, and axonal injury to, RGCs is important in retinal neuropathy. Similar to CNS neurons in other neurodegenerative diseases, death of RGCs is irreversible and can directly disturb visual pathway signal transmission, resulting in impaired visual function. Glaucoma is a retinal neurodegenerative disease characterized by the loss of, and axonal injury to, RGCs. Therefore, protection of RGCs is important in the treatment of retinal neuropathies such as glaucoma. In this study, we used the ONC model to explore the mechanism(s) underlying the loss of RGCs that occurs during the development of glaucoma.

During the course of nerve injury, the myelin‐associated inhibitory protein LINGO‐1 mediates neuronal survival and axonal regeneration, thus contributing to neurodegeneration.^7^ LINGO‐1 is expressed in neurons and oligodendrocytes of the CNS and is significantly upregulated in various neurological disorders such as Parkinson's disease, multiple sclerosis, and nerve injuries (eg, spinal cord injury).^6^, ^10^, ^15^, ^32^ Blocking LINGO‐1 function promotes the survival of neurons and axon regeneration after nerve injury.^12^ Notably, we and others have found that inhibition of LINGO‐1 promotes the survival of RGCs and axon regeneration after injury to the optic nerve.^19^, ^31^ Similarly, in the present study, LINGO‐1 expression was upregulated in the ONC‐injured retina. Furthermore, LINGO‐1 promoted the apoptosis of RGCs cultured under serum‐deprived conditions.

In view of the important pathophysiological role of LINGO‐1 in mediating the death of RGCs, we investigated the upstream regulatory factors. In microarray analyses, we found that SP1 was upregulated in the RGCs of ONC‐injured retinas, and LINGO‐1 promoter analyses revealed that SP1 increased the expression of LINGO‐1 by binding to its promoter region. Furthermore, SP1 knockdown antagonized LINGO‐1–induced apoptosis of RGCs under serum‐deprivation conditions. In addition, SP1 and LINGO‐1 were simultaneously upregulated in injured RGCs. These data imply that SP1 regulates LINGO‐1 expression at the transcriptional level and that upregulation of SP1 is implicated in LINGO‐1–mediated death of RGCs. Indeed, SP1 has been reported to play important pathophysiological roles in a variety of neurodegenerative diseases, including Alzheimer's, Huntington, and Parkinson's diseases, and to regulate disease‐related pathogenic genes such as APOE, LRRK2, and P16INK4A.^22^, ^23^, ^33^, ^34^ This abnormal upregulation of SP1 expression in neurons is attributed to inflammation and oxidative stress in the surrounding environment.^35^, ^36^ These assumptions are in line with the consensus on the pathogenesis of glaucoma, a neurodegenerative disease.^37^, ^38^ High intraocular pressure leads to inflammatory and oxidative stress responses by Müller cells and glial cells, resulting in loss of RGCs and axonal damage.^39^, ^40^ Likewise, the biological processes involved in the subsequent axonal injury (inflammatory response, oxidative stress, and so forth) also promote the death of RGCs.^41^ Moreover, SP1 regulates the expression of the RGC‐specific gene *syt11*,^42^ and its expression is upregulated in the RGCs of patients with diabetic retinopathy.^43^, ^44^ Collectively, the data indicate that SP1 is involved in LINGO‐1–mediated death of RGCs in the ONC‐injured retina.

We evaluated the role of SP1 in the LINGO‐1–mediated death of RGCs. Intravitreous injection of AAV2‐SP1 shRNA was performed at 2 weeks before ONC to knock down SP1 expression in RGCs; this resulted in reduced expression of LINGO‐1. To investigate the neuroprotective effects of inhibition of SP1, we used OCT to monitor RNFLT and assess the axons of RGCs. Inhibition of SP1 promoted survival of RGCs and increased RNFLT, consistent with our previous report of the neuroprotective effects of LINGO‐1 antagonism.^19^

We then evaluated the protective effects of inhibition of SP1 on visual function. Surprisingly, inhibition of SP1 did not significantly modulate N1‐P1 amplitude or N1 latency. The two major possible explanations for these conflicting findings are that inhibition of SP1 preserved the structure of RGCs but did not restore visual function. First, inhibition of SP1 may not protect the subcellular organelles of RGC axons from damage such as disintegration of the synaptic assembly or may fail to promote synapse repair after damage. BDNF, CTNF, and NT3, among other factors, promote neuronal survival and synaptic regeneration after injury.^45^, ^46^, ^47^ Thus, protection of RGCs may, in addition to myeloid inhibitors, require application of neurotrophic components. Second, because visual stimulation can promote axon regeneration and recovery of visual function,^48^ enhancement of neural activity may increase the efficacy of LINGO‐1–based neuroprotective therapy. In addition, neuronal function and survival are very sensitive to mitochondrial dysfunction. Recently researches have shown that mitochondrial dysfunction is closely associated with retinal neuronal damage, which can be remedied by stem cell‐mediated mitochondrial repair ^49^, ^50^ Thus, we suppose that neurotrophic treatment, visual stimulation, and mitochondrial transplantation should be incorporated in integrated therapeutic strategy for promoting retinal neuronal repair. Technical concerns also need to be taken into consideration; further studies involving a longer follow‐up period and more comprehensive evaluation of visual function are warranted.

We report the upregulation of SP1 and LINGO‐1 expression in RGCs in the ONC‐injured retina. In vitro, SP1 regulated LINGO‐1 expression at the transcriptional level and promoted the LINGO‐1–mediated death of RGCs. Furthermore, SP1 knockdown had a neuroprotective effect in vivo. Our results provide important insight into the mechanism of LINGO‐1–mediated death of RGCs in patients with glaucoma. Further studies are required to increase our understanding of the mechanism(s) of RGC‐related synaptic damage and formulate a neuroprotective strategy for glaucoma involving stimulation of the visual system.

## CONFLICT OF INTEREST

The authors declare no conflict of interest.

## Supporting information

Fig S1
