# Supplementary figures and images for: SP1‐mediated upregulation of LINGO‐1 promotes degeneration of retinal ganglion cells in optic nerve injury
Source: CNS Neurosci Ther. 2020 Jun 19;26(10):1010–20. doi: 10.1111/cns.13426 (PMC7539844; doi:10.1111/cns.13426)

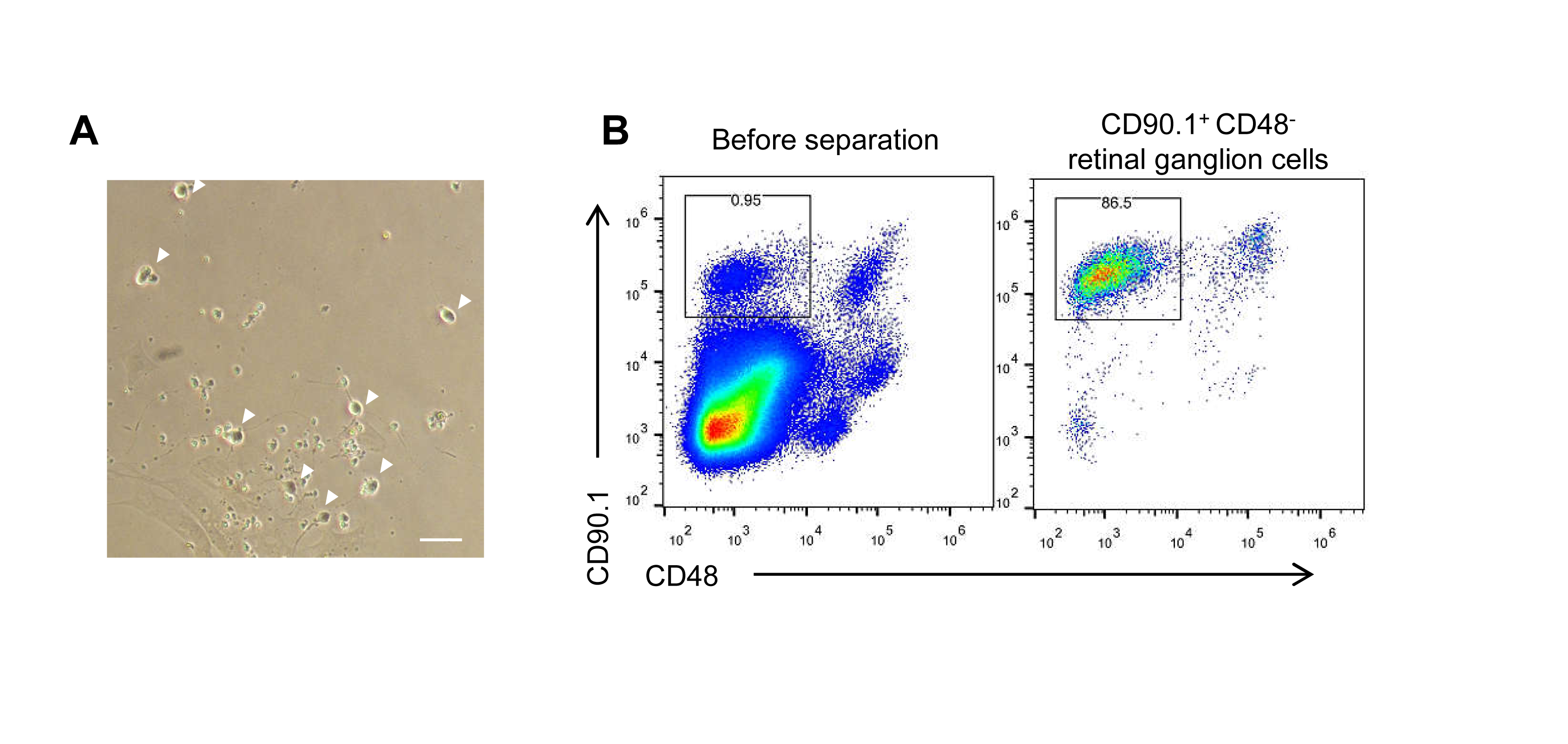

Supplement: Supplementary file 1 — Fig S1 [file CNS-26-1010-s001.tif]
